# Purtscher-Like Retinopathy Associated with Anorexia Nervosa

**DOI:** 10.1155/2016/1934091

**Published:** 2016-03-16

**Authors:** Bugra Karasu, Betul Onal Gunay, Gurkan Erdogan, Esra Kardes, Murat Gunay

**Affiliations:** ^1^Umraniye Training and Research Hospital, Department of Ophthalmology, 34764 Istanbul, Turkey; ^2^Zeynep Kamil Maternity and Children's Disease Training and Research Hospital, Department of Ophthalmology, 34668 Istanbul, Turkey

## Abstract

A 21-year-old girl presented with acute painless vision loss in her right eye. There was no remarkable ocular history and she had a history of anorexia nervosa. At presentation best-corrected visual acuities were counting fingers from 2 meters and 20/20, in the right and left eyes, respectively. Slit lamp examination result was normal. Fundus examination revealed multiple cotton wool spots and intraretinal hemorrhages surrounding the optic disc and macula in the right eye. Fluorescein angiography showed capillary filling defect and leakage from optic disc in the late phase of the angiogram. One week later best-corrected visual acuities remained the same in both eyes with similar fundus appearance. One month after initial presentation visual acuity was 20/20 in both eyes with no abnormality in fundus appearance.

## 1. Introduction

Purtscher's retinopathy (PR) was firstly defined by Otmar Purtscher in 1910 in a patient following head trauma. Fundus examination in both eyes displayed various sizes of cotton wool spots with intraretinal hemorrhages limited to posterior pole which were named* Purtscher flecken* [1, 2]. Besides traumatic PR, this characteristic fundus view has been found in several disorders including acute pancreatitis, hemolysis, elevated liver enzyme levels and low platelet levels (HELLP) syndrome, hemolytic uremic syndrome, amniotic fluid embolism, thrombocytopenic purpura, and renal failure in which all were named Purtscher-like retinopathy [3, 4]. Studies indicated the rate of PR or Purtscher-like retinopathy as 0.24 cases per million per year [5].

Anorexia nervosa (AN), a kind of eating disorder, is a psychological disease that leads to ruinous physical consequences. Patients with AN restrict their food intake and consumingly lose weight [6]. No report exists so far regarding the presence of Purtscher-like retinopathy in AN. Herein, we presented clinical characteristics of a patient with Purtscher-like retinopathy due to AN in the current report.

## 2. Case Presentation

A 21-year-old girl was admitted to the outpatient eye clinic of Umraniye Training and Research Hospital with acute painless vision loss in her right eye. The patient had a history of AN. In ophthalmologic evaluation, best-corrected visual acuity (BCVA) was counting fingers from 2 meters and 20/20, in the right and left eyes, respectively. No significant finding was observed on anterior segment examination in both eyes. Intraocular pressure was 15 mm-Hg in both eyes. No relative afferent pupillary defect was present.

**Figure 1:**
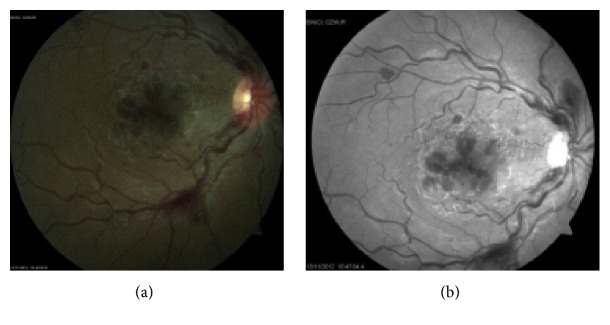
Initial examination of the right eye: (a) fundus photography; (b) red free fundus photography.

Fundus examination revealed multiple cotton wool spots and intraretinal hemorrhages surrounding the optic disc and macula in the right eye (Figures 1(a)-1(b)).

**Figure 2:**
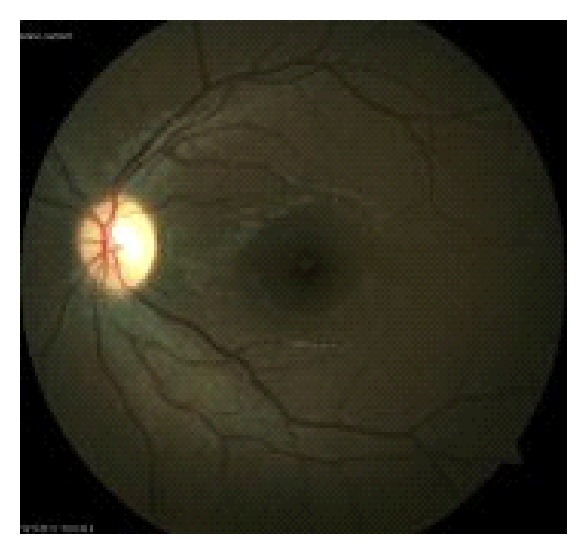
Fundus photograph of the left eye at initial examination.

No abnormality was observed in the left eye (Figure 2).

**Figure 3:**
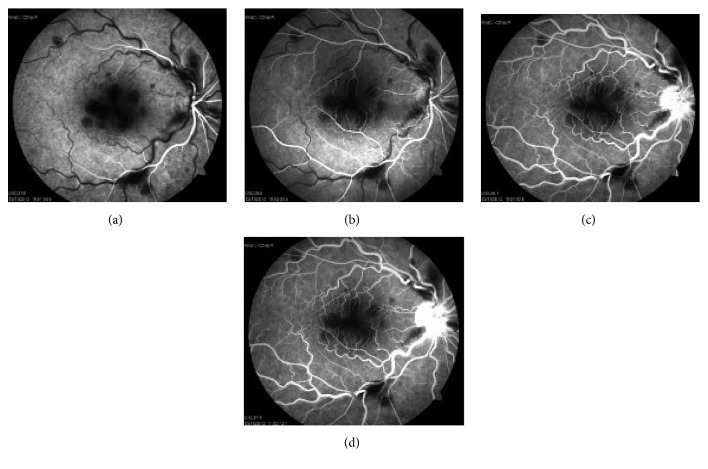
Fundus fluorescein angiography of the right eye at initial examination. (a and b) Early phase of FFA shows capillary filling defect. (c and d) Late phase of FFA shows leakage from the optic disc.

Fundus fluorescein angiography (FFA) was considered. The FFA analysis showed capillary filling defect (Figures 3(a) and 3(b)) and leakage from optic disc in the late phase of the angiogram (Figures 3(c) and 3(d)).

Further investigation was performed. The rheumatoid factor, antinuclear antibody, anti-antibodies to extractable nuclear antigen, anti-neutrophil cytoplasmic antibody, and anti-Jo1 antibody were all found to be negative. Carotid Doppler imaging and cranial magnetic resonance imaging were normal. No other abnormality was present after laboratory investigation. Serum albumin (4.5 g/dL), aspartate aminotransferase (17 IU/L), alanine aminotransferase (16 IU/L), serum amylase (75 IU/L), lipase (47 IU/L), *γ*-glutamyl transpeptidase (30 IU/L) and lactate dehydrogenase (168 IU/L) levels were normal. Also systemic evaluation revealed no other abnormality. The diagnosis of Purtscher-like retinopathy was made. Clinical observation was considered.

**Figure 4:**
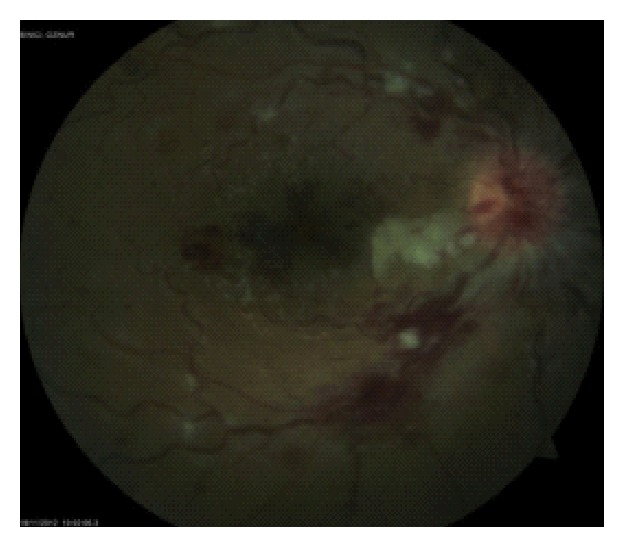
Fundus photograph of the right eye one week after the initial examination.

One week after the initial examination, BCVA remained the same in both eyes with similar fundus appearance (Figure 4).

**Figure 5:**
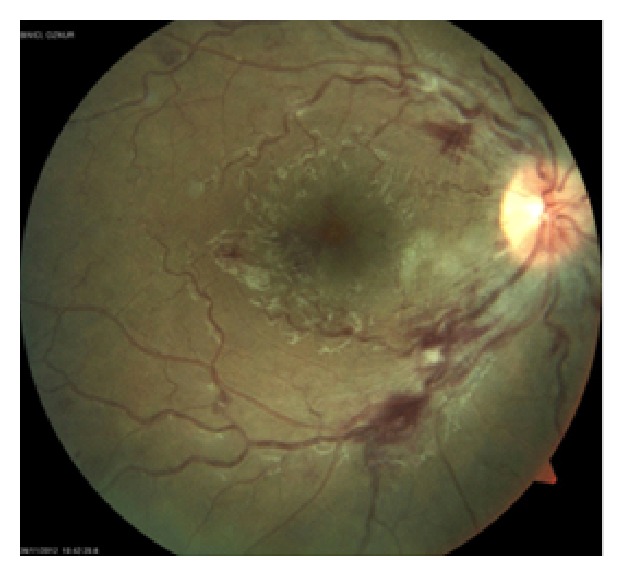
Fundus photograph of the right eye two weeks after the initial examination.

Two weeks later, her vision improved to 20/33 in the right eye. The number and density of the cotton wool spots and retinal hemorrhages decreased (Figure 5).

**Figure 6:**
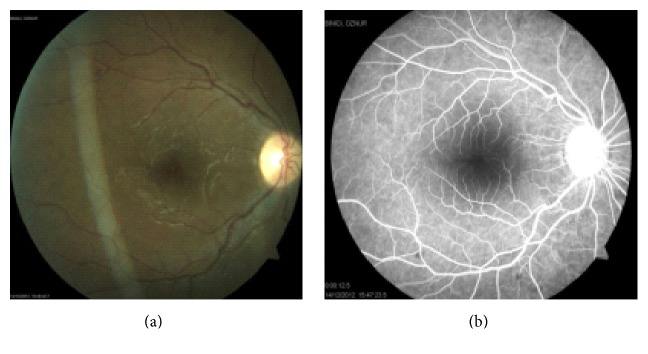
Fundus photograph (a) and FFA (b) of the right eye at the last visit.

At the last visit, one month after presentation of the retinopathy, during the ophthalmologic examination, the BCVA was 20/20 in both eyes with no abnormality in fundus appearance (Figures 6(a)-6(b)).

## 3. Discussion

Following the description of Purtscher retinopathy in a traumatic case by Otmar Purtscher in 1910, studies have identified similar clinical findings to this disease in several systemic disorders in which it has been called Purtscher-like retinopathy. The well-known characteristic appearance of this condition includes multiple areas of cotton wool spots in posterior pole in company with papilloedema and patchy intraretinal hemorrhages [7, 8].

Fundus fluorescein angiographic studies have observed retinal capillary leakage around the fovea with capillary filling defect and retinal whitening. All these observations have been associated with reduction in VA [9].

Supporting the abovementioned findings, at initial visit, fundoscopic appearance of our case demonstrated multiple cotton wool spots and intraretinal hemorrhages around the optic disc and macula with reduced VA. Furthermore, capillary filling defect and leakage from optic disc in the late phase of the angiogram were also detected.

No proven specific effective treatment has been suggested for Purtscher or Purtscher-like retinopathy [10]. Improvement of vision has been reported following systemic methylprednisolone treatment [11].

To the best of our knowledge, we reported Purtscher-like retinopathy in a case with AN for the first time. Although management with systemic steroids may have beneficial effect on VA recovery, our case showed a BCVA of 20/20 one month after the initial presentation without any treatment.
